# Algebraic Distribution of Segmental Duplication Lengths in Whole-Genome Sequence Self-Alignments

**DOI:** 10.1371/journal.pone.0018464

**Published:** 2011-07-14

**Authors:** Kun Gao, Jonathan Miller

**Affiliations:** Physics and Biology Unit, Okinawa Institute of Science and Technology, Kunigami, Okinawa, Japan; American Museum of Natural History, United States of America

## Abstract

Distributions of duplicated sequences from genome self-alignment are characterized, including forward and backward alignments in bacteria and eukaryotes. A Markovian process without auto-correlation should generate an exponential distribution expected from local effects of point mutation and selection on localised function; however, the observed distributions show substantial deviation from exponential form – they are roughly algebraic instead – suggesting a novel kind of long-distance correlation that must be non-local in origin.

## Introduction

The basic mechanisms of genome sequence evolution include point mutation, insertion and deletion, and recombination; in particular, segmental duplication [1]. The fundamental importance to evolution of gene duplication was stressed in Ohno's classic text, “Evolution by Gene Duplication” [2], but had been appreciated since the early 1900's – well before the discovery of DNA [3]. Over the last twenty years, advances in genome sequencing technology have confirmed what before could only have been inferred by tedious experimentation and a duplication can now be read directly from a whole-genome sequence.

The mechanisms and impact of sequence duplication have received great attention over the years; for reviews, see [1]. Both selective and neutral mechanisms are believed to be important, but their roles are not always easy to tease apart: concerted evolution can yield sequence homogenization that might be readily misattributed to selection [4], [5].

The length distribution of exact and nearly-exact sequence duplications within a single genome is characterized for the first time in this manuscript, and certain properties that appear to be general to the genome sequences of a diverse set of species are identified – specifically a “heavy,” roughly algebraic tail for long sequences, that we call “ultra-duplication.” This observation recasts the interpretation of long-range sequence correlations, first described twenty years ago, by exhibiting an independent measure that could make it possible to distinguish among competing models for this phenomenon.

### Sequence duplication

Broadly speaking, duplications are classified into “whole-genome duplications,” for which good evidence has been demonstrated in a number of bacterium, plant and vertebrate genomes, and “segmental duplications” (SD), which are common and involve sequences that are much shorter than whole genomes. Our focus here is on SD, which have been intensively studied for almost a century. Thus, Bailey *et al.* observed in 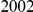 that recent SD, defined as sequence pairs longer than 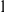 kb (kilobase) with at least 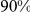 identity, account for some 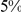 of the human genome [6] and are often involved in chromosome rearrangements underlying genetic disease. Subsequently Cheung *et al.* computed that around 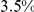 of the approximately 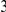 gigabase human genome consists of SD, defined as at least two sequences longer than 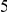 kb and sharing more than 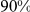 identity. Patterns of SD were further characterized by Zhang *et al.* [7].

The *formation* of SD is customarily regarded as a largely neutral process, i.e., independent of any function of the duplicated sequence. Exceptions are duplications of self-replicating elements, such as SINES, LINES, complex repetitive interspersed sequences, transposons, and so on – but these sequences are for the most part excluded from our analysis of eukaryotic genomes by repeat-masking. SD is believed generally to involve replication of a sequence as an integral unit: it is thought to be relatively improbable that a long sequence copy will have been created by the concatenation of two shorter non-overlapping sequences that evolved separately and independently. The *preservation* of sequence identity once the duplicate is created, is another matter; selective and neutral processes become involved, whose effects are not always readily disentangled.

A further distinction is sometimes made between SD and “copy number variants” (CNV). Copy number variants are sequences that occur in different numbers within different individuals of a population. If the genome of a single individual is the only sequence available, it is unclear how to distinguish between SD and CNV. The studies described here involve genome assemblies that are – in principle – supposed to reflect the (possibly haploid) genome sequence of a single individual. It is not clear whether existing assembled genome sequences of additional individuals within any single vertebrate species are yet of sufficient quality to study duplication genome-wide, because often duplications pose the greatest challenge to the genome assembly process. Therefore, for our purposes any duplicated sequence will be called an SD; on the other hand, CNV suggests that some contribution to what we call SD arose from recent duplication events.

A wide variety of paths to sequence duplication have already been discovered; the precise mechanisms of some of these have been characterized in great detail, and sometimes they exhibit intrinsic length scales. On the other hand, from the genome sequence of a single individual it can be difficult to infer the mechanism of origin of any given duplication with much certainty, and we may not yet be aware of all pathways for sequence duplication.

Therefore it is necessary to distinguish among (i) mechanisms of sequence duplication; (ii) the proportion of sequence duplications attributable to any given mechanism; and (iii) the impact of sequence duplication on the genome as a whole. It may be possible to usefully and productively characterize each of these items, (i)–(iii), separately without necessarily having any understanding of the relations among them. This paper focuses on (iii), with the hope that once the impact of sequence duplication on the genome as a whole is worked out, the chief contributing mechanisms can be tracked down exhaustively.

Finally, the pivotal role of DNA repair in the processes of duplication and recombination can't be overestimated. Because our focus here is their net impact upon genome sequence evolution, it is convenient in this context to apply the terms “duplication” and “recombination” loosely so as to encompass effects of repair mechanisms and gene conversion. In other contexts, such usage could be misleading.

### Ultraduplication

Our primary object of study is the distribution of duplicated sequence lengths: Given a *single* genome, for each length (in bases or nucleotides) 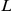, we count how many sequences of length 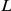 occur more than once, 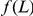. Our interest in this function is that for a chromosome-size random sequence generated by a local dynamics, it ought to take an exponential form. A deviation from an exponential could suggest the action of selection or of a non-local neutral process.

Recently, it was observed that the length distribution of sequences strongly conserved among sufficiently divergent genomes is generally (approximately) algebraic in form. The latter class includes (but is not limited to) the so-called “ultraconserved” sequences. We conjectured that this observation implicated neutral processes, such as recombination, in the evolution of strongly conserved sequences, whose effects could require a recalibration of standard comparative genomics methods that rely on a null model of uncorrelated local mutations to infer selection from sequence conservation.

In this manuscript, we compute the distribution of duplicated sequence lengths for a variety of chromosomes and genomes, and demonstrate that it too is approximately algebraic. We have termed this phenomenon, “ultraduplication.”

Out of concern for assembly errors and to exclude potentially uncharacterized transposons and retroelements, studies of SD in eukaryotes have often been limited to duplications that are longer than 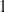 to 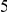 kb and of greater than 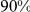 sequence identity [1]. The latter concern, we address by studying repeat-masked sequence only, and by illustrating the contribution of functional coding Hox gene sequences to the distribution. The algebraic character of prokaryotic duplicated sequence length distributions argues against the former concern, as many prokaryote genomes are believed to have been obtained with high accuracy. Therefore, we eliminate any explicit restriction on length, and explore a systematic reduction of stringency on sequence identity.

We perform self-comparisons for several genomes by heuristic, but standard, genome *alignment* methods; however, our principal conclusions have been confirmed and extended by exhaustive all-on-all genome self-comparison – 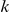-mer self-*intersection* – which we describe elsewhere, is completely independent, and involves no heuristics [8]. Alignment and intersection can be thought of as complementary tools, each with their own strengths and weaknesses. For comparisons between or among different genomes, these tools yield more-or-less consistent outcomes wherever their applicability overlaps, a consistency also shared between self-alignment and self-intersection.

We find that length (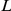) distributions of duplicated sequences, 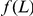, like those of conserved sequences, take a roughly algebraic (or power-law) form for large 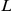, that can be usefully parameterized by an exponent 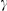: 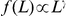. For eukaryotic genomes, conserved sequences typically show 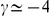, while duplicated sequences exhibit 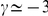 (typically between 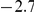 and 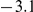). For prokaryotic genomes, exponents tend to be larger in magnitude and vary more widely.

**Figure 1:**
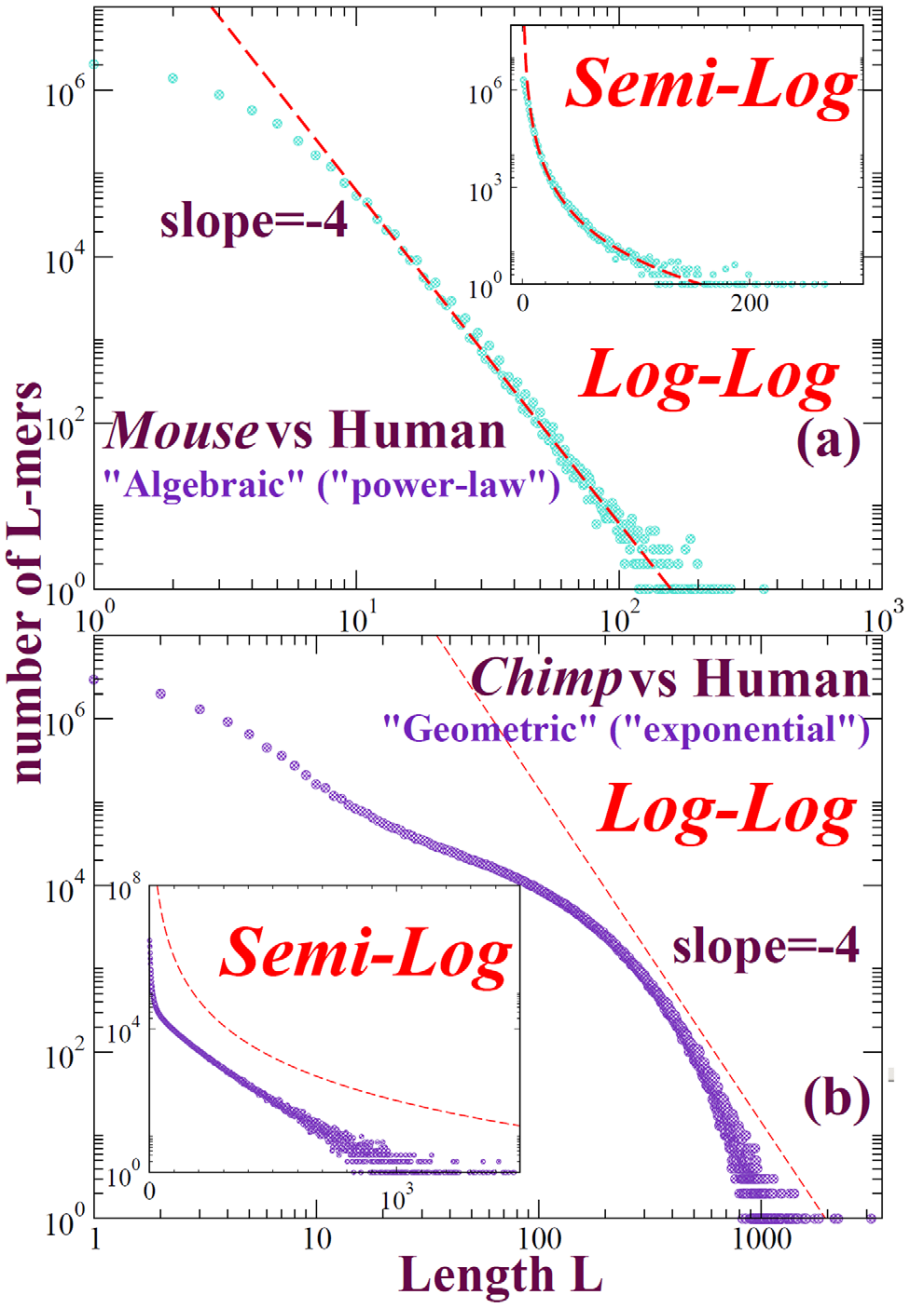
Length distributions of perfectly conserved sequences from natural genome alignments typically yield power laws, as shown in subfigure A for mouse/human alignment – *provided that the genomes are not too closely-related*, as illustrated by subfigure B the approximately exponential length distributions from chimpanzee/human alignment. Relaxing the matching condition, so that A = G and C = T for example, yields substantially more aligned sequence, yet shapes that are very similar overall to those shown here [8], [33].

In this manuscript, the distinction that we draw between algebraic and exponential is indicated by Figure 1: except at short lengths, the curves are straighter on a log-log plot than on a semi-log plot, or *vice versa*. Some validation of this point of view is provided by the subsection on scale-free duplication dynamics in the Text *S1*; however, **if the reader prefers to think of the terms “power-law” (or “algebraic”) and “exponential” (or “geometric”) merely as qualitative labels for the shapes illustrated in** **Figure 1** **, it will be sufficient for our purposes.** Finer distinctions are obtained elsewhere and are not intended here; in section **VII.A** we place our observations into the general context of power-law distributions.

Within single genomes or chromosomes, we characterize the length distribution of “contiguously matched runs” (CMRs) – continuous uninterrupted runs of matching bases subject to one of several criteria given explicitly below. No assumptions are made about origin or function. We compute matched runs by pairwise alignment methods, and study a variety of genomes to ascertain the generality of the power-law. The relevance of our global, genome-wide statistics to local sequence characteristics is illustrated by elucidating these statistical features within the human and mouse Hox gene clusters.

## Results

### Contiguously matched runs (CMRs) in the alignment

Given an alignment, we study its CMRs – continuous uninterrupted runs of matching bases – subject to one of the following matching criteria, in order of decreasing stringency:

- Exact matches: Each of the four nucleotides (A,T,G,C) matches itself only; a mismatch or indel terminates a run of matches;
- A = G, C = T: In addition to the exact matches, A and G, C and T are also matched pairs; an indel or any mismatch involving other than an A/G or T/C pair terminates the run;
- Indel-terminated matches: Any nucleotide matches any other; only an indel terminates the run;
- Alignment blocks: Alignment blocks are fragments with high similarities relative to their neighborhood, returned by the alignment procedure; they can be thought of for convenience as “paragraphs” composing the alignment. They span exact matches, mismatches and indels.

We also count contiguous indels (gaps) appearing in either the query sequence or the target sequence. Apparently, the contiguous indel set complements the indel-terminated match set within each alignment block.

Matching criteria 1 through 4 successively relax the matching condition. CMRs counted according to a tighter criterion are always contained within those counted according to a more relaxed criterion. Therefore, locally within an alignment block, CMRs counted according to different critera exhibit a nested or hierarchical structure. Figure *S1* illustrates the corresponding nesting structure in the self-alignment of *Anabaena variabilis* whole-genome (see supporting figures).

### Basic properties of CMR length distributions

**Figure 2:**
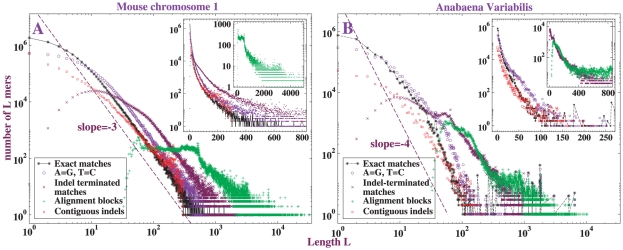
Length distributions of the CMRs counted by different matching criteria. A: CMRs in mouse chromosome 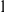 self-alignment computed by Blastz-Raw; B: CMRs in *Anabaena variabilis* whole-genome self-alignment computed by Blastz-Raw. The reference lines have fixed slopes of 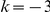 and 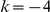 on the log-log plot. The insets show same data on semi-log plots.

**Figure 3:**
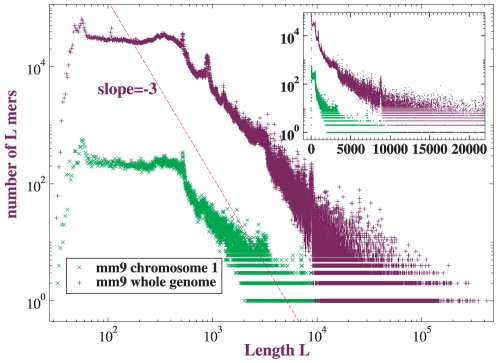
Expansion of the length distribution of alignment blocks in mouse self-alignment. Two different curves show mouse chromosome 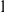 and mouse whole-genome self-alignments, respectively. Inset shows same distributions on a semi-log plot. Alignments computed by Blastz-Raw.

Figure 2 shows the length distributions of the CMRs in the Blastz-Raw self-alignments of a eukaryotic sequence (mouse chromosome 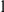) and a prokaryotic sequence (*Anabaena variabilis* whole-genome). Within each subfigure, CMRs are counted by each of the respective matching criteria. In Figure 2 A, the length distributions of CMRs from mouse chromosome 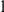 self-alignment, it is evident that:

- All the distributions have power-law shapes over a substantial range. The exponents are close to 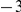.
- Outside of this range, the distributions deviate from power-law. Such deviations are inevitable and may be attributable at short lengths to the intrinsic scale of a single nucleotide and at large lengths to the finite length of the genome or chromosome, or to artifacts of incomplete or immature assembly. The former finite-size corrections are standard in examples of scaling in physics and a complete theory must account for them. In whole-genome sequence data the length distribution of assembled contigs often exhibits a sharp peak at a scale on the order of a few thousand bases; in subsequent versions of the assembly, when available, this scale increases and the quality of the power-law improves.
- Except for the alignment blocks, length distributions of the CMRs arguably have a power-law character because they are linear on the log-log plot but upward concave on the semi-log plot in the large insets. A power-law appears to be more suitable as the matching criterion becomes tighter.
- In addition to A = G,C = T CMRs, for mouse chromosome 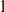 we also studied A = C,G = T and A = T,G = C CMRs. They differ inappreciably from exact matches (see Figure *S2*). A similar observation was reported for inter-genome comparisons [8], and we believe it applies generally. Possible origins of the difference between A = G,C = T matches and the other two include transition-transversion asymmetry and biased gene conversion.
- The length distribution of alignment blocks does not conform to a power-law as well as the others. Alignment blocks are longer than other CMRs, and the expanded semi-log plot in the smaller inset exhibits the curvature clearly. Since alignment blocks are the most coarse-grained CMRs, greater finite-size effects might be anticipated, and they can be confirmed by plotting the corresponding distribution for mouse *whole-genome* (rather than chromosome 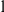 only) self-alignment as in Figure 3. The whole-genome contains an order-of-magnitude more sequence than the largest chromosome, and its alignment evidently fits a power-law over a larger range than single-chromosome alignment. Nevertheless, the shapes of the distributions for these two alignments are qualitatively similar, and the length distribution of alignment blocks appears to be better recapitulated by a power-law than an exponential.

Bacteria genomes are much shorter than vertebrate genomes, so finite-size effects may be correspondingly greater. There are fewer simple and tandem repeats in bacterial than in eukaryotic genomes, and they are not usually repeat-masked. These distributions exhibit stronger fluctuations, and the powers tend lie around 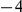 (except the contiguous indels, which still lie between 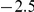 and 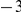). For indel-terminated runs and alignment block lengths, it's hard to ascertain whether their length distributions are power-law or exponential, but for the other three curves, comparing the log-log plot and the semi-log plot is suggestive of a power-law. Potential finite-size effects can be investigated more directly in a model for gene duplication that can be shown to yield a power-law distribution asymptotically in chromosome length, and the comparison of shapes is quite favorable; see Text *S1* and Figure *S3*. Thus, length distributions of CMRs from bacteria self-alignment qualitatively resemble those of vertebrates.

Limited data on the length distribution of contiguous insertions and deletions less than around 60 bases long were obtained in support of an algebraic gap length distribution within certain special genomic regions, such as pseudogenes [9]–[11]. The calculations reported here generalize this observation significantly over length and species.

### Insensitivity of the power-law to the alignment method

**Figure 4:**
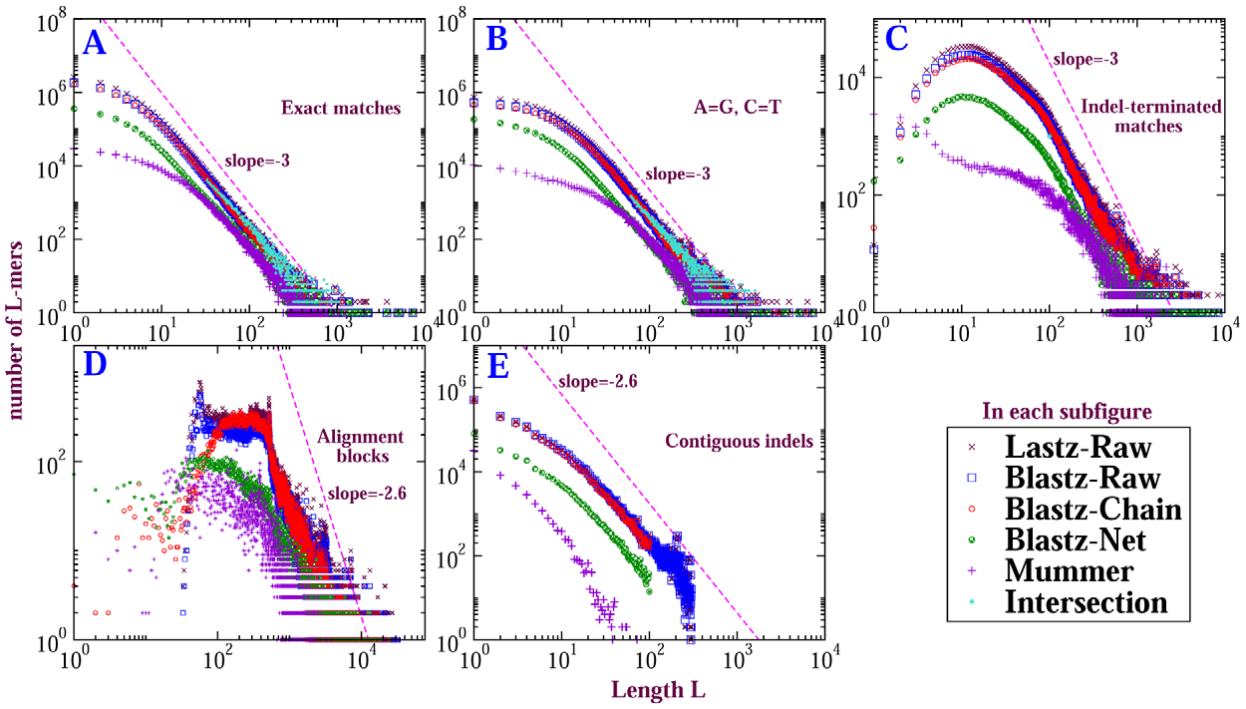
Length distributions of CMRs from self-alignment of mouse chromosome 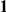 computed by different methods. Subfigures exhibit CMRs for different matching criteria. In contrast to the distribution for A = G,C = T matches, which is shifted significantly rightward from the exact matches, distributions for A = C,G = T and A = T,G = C matches differ inappreciably from exact matches; they are illustrated in Figure *S2*.

Sequence alignment algorithms involve heuristics that could produce artifacts. We performed the self-alignments by different methods and compared the length distributions generated by each of them. Figure 4 displays length distributions of CMRs from mouse chromosome 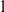 self-comparison computed by sequence *intersection* and by five alignment methods: Lastz-Raw, Blastz-Raw, Blastz-Chain, Blastz-Net and Mummer. Released while our study was underway, Lastz (http://www.bx.psu.edu/r~sharris/lastz/) is an improved version of Blastz; however, Mummer is independent of the Lastz family. In the figure, it is apparent that the length distributions agree with one another qualitatively. The differences among them (discussed in Text *S2*) are for our purposes minor. Comparison of dot plots (indicating spatial arrangement of the CMRs) for these different alignment methods also yields only minor differences (see Figure *S4*).

### Similarity of length distributions among mouse chromosomes

So far, we have exhibited length distributions from Blastz-Raw self-alignments of mouse chromosome 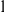 and *Anabaena variabilis* whole-genome. Figure *S5* shows the length distributions of exact matches in the Blastz-Raw self-alignments for all mouse chromosomes. Apart from the 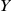 chromosome, they qualitatively resemble mouse chromosome 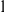, with exponent between 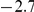 and 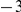.

### Similarity of length distributions among a variety of species

**Figure 5:**
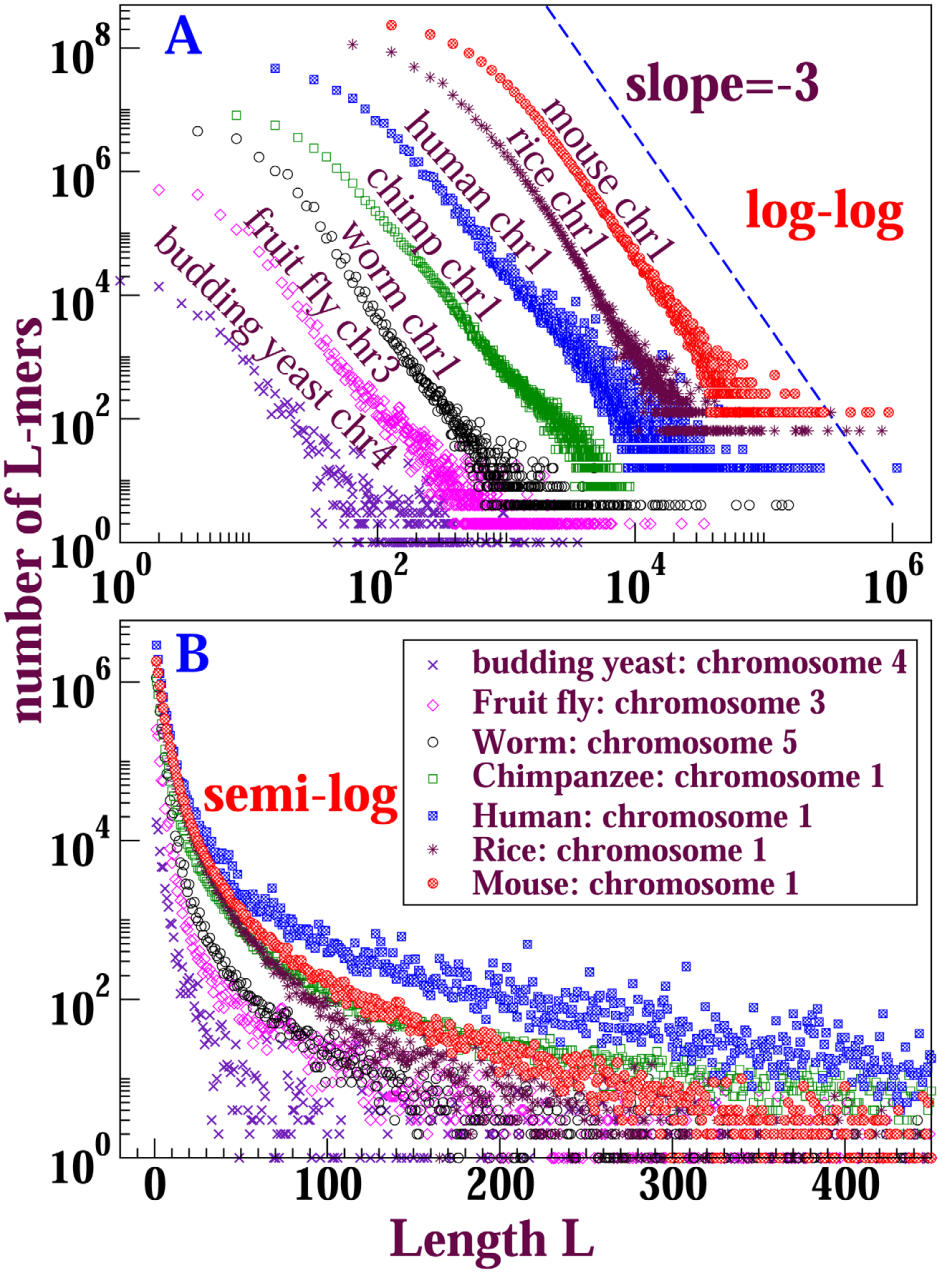
Length distributions of exact matches from Blastz-Raw self-alignments for different eukaryotic species. For each species, we self-aligned its longest chromosome. The upper figure shows the log-log plot, the lower a semilog plot for the same distributions. In order to show all the curves clearly, we translated the curves in the log-log plot by factors: 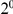 for budding yeast, 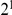 for fruit fly, 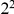 for worm, 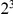 for chimp, 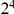 for human, 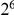 for rice and 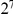 for mouse. Both x-values and y-values are multplied by the respective factor.

**Figure 6:**
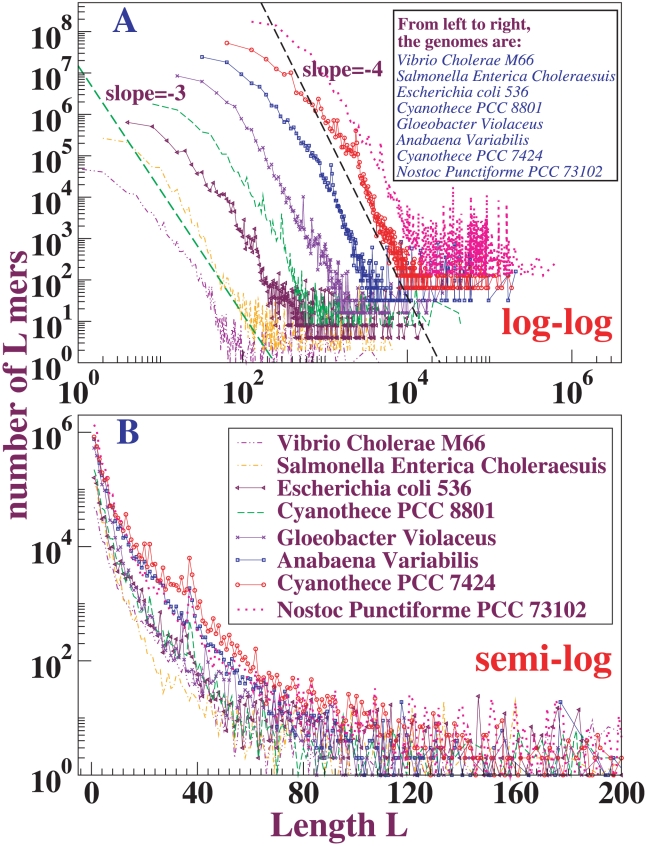
Length distributions of exact matches in Blastz-Raw self-alignments for different prokaryotic species. We align the single largest chromosome (usually there is only one chromosome) and omit any plasmids. The upper figure shows the log-log plot; the lower a semilog plot for the same distributions. In order to show all the curves clearly, we translated curves in the log-log plot by factors: 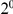 for *Vibrio Cholerae M66*, 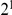 for *Salmonella Enterica Choleraesuis*, 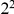 for *Escherichia coli 536*, 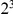 for *Cyanothece PCC 8801*, 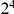 for *Gloeobacter Violaceus*, 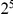 for *Anabaena variabilis*, 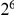 for *Cyanothece PCC 7424* and 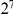 for *Nostoc Punctiforme PCC 73102*. Both x-values and y-values are multplied by the respective factor.

In Figures 5 and 6, we plot length distributions of exact matches from Blastz-Raw self-alignments of several chromosomes, respectively eukaryotic and prokaryotic. For each eukaryotic genome, we obtained the soft repeat-masked sequence of the longest chromosome from the Ensembl database; for bacteria, we simply use their whole-genomes directly. Many of the curves fall directly on top of one another; in order to show the distributions clearly on log-log plots we translated each curve as indicated in the figure captions. The eukaryotes show power-law distributions, with the powers quite close to 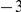. For bacteria, the distributions fluctuate more strongly; however, they plausibly have power-law regimes with exponents mostly between 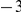 and 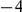. Thus it appears that power-law length distribution is a general feature of the genomes of a wide range of species. From now on focus on mouse chromosome 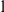 and *Anabaena variabilis* whole genome for detailed characterization.

### Forward and Backward Alignment; Projection

In Text *S3*, we illustrate power-law length distributions among different subsets of the alignment. We observe that forward and backward alignments qualitatively resemble one another and the full alignment (see Figure *S6*). We also project the dot-plot onto the chromosome in order to determine the total number of bases covered by the aligned sequences, which may overlap one another. This process yields runs of chromosomal sequence, each base of which is contained within some aligned sequence. The length distribution of these runs is also seen to be algebraic (see Figure *S7*).

### Self-alignment and inter-genome alignment among Hox genes

The above discussion applies to global (whole-genome or whole-chromosome) alignment. In fact, an algebraic form of the length distribution is not solely a global feature, but is also satisfied locally. In this section, we investigate the properties of Hox (homeobox) gene sequences within whole-chromosomal alignments. Transcription factors that play essential roles in development, Hox proteins tend to be strongly conserved. Typically large numbers of Hox genes are arranged in several clusters within a single genome; for example, mouse contains 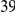 Hox genes comprising 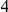 clusters. It is believed that these Hox genes arose from ancient duplications [12]. We demonstrate that Hox genes *by themselves* exhibit algebraic distributions of duplicated sequence lengths whose shapes are similar to genome-wide length distributions.

Our operational definition of a Hox gene is taken as the sequence between start and end coordinates of a Hox protein-coding gene in the Ensembl whole-genome sequence database Version 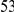; it includes introns, exons, UTRs, and protein-coding sequences. Aligned fragments, in which both the query and target sequence are contained within a Hox gene (although not necessarily the same Hox gene), were eliminated from (i) human self-alignment; (ii) mouse self-alignment; and (iii) human/mouse alignment. CMRs were counted within each of these three sets. Figure *S8* illustrates this procedure: (i) for each species we obtain all Hox gene-containing chromosomes (chromosomes 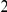, 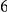, 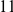 and 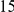 of mouse each contain Hox clusters comprised of multiple Hox genes); (ii) the chromosomes are aligned pairwise; (iii) CMRs are extracted from alignment fragments that are fully contained by Hox genes.

Figure *S8* depicts the 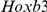/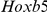 fragments retrieved from the self-alignment of chromosome 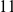, with dashed rectangles and arrows indicating the steps of this expansion; the nesting of CMRs counted with different stringencies is also indicated. Nearly all alignment blocks contain a homeobox domain protein-coding sequence, indicated in the figure.

**Figure 7:**
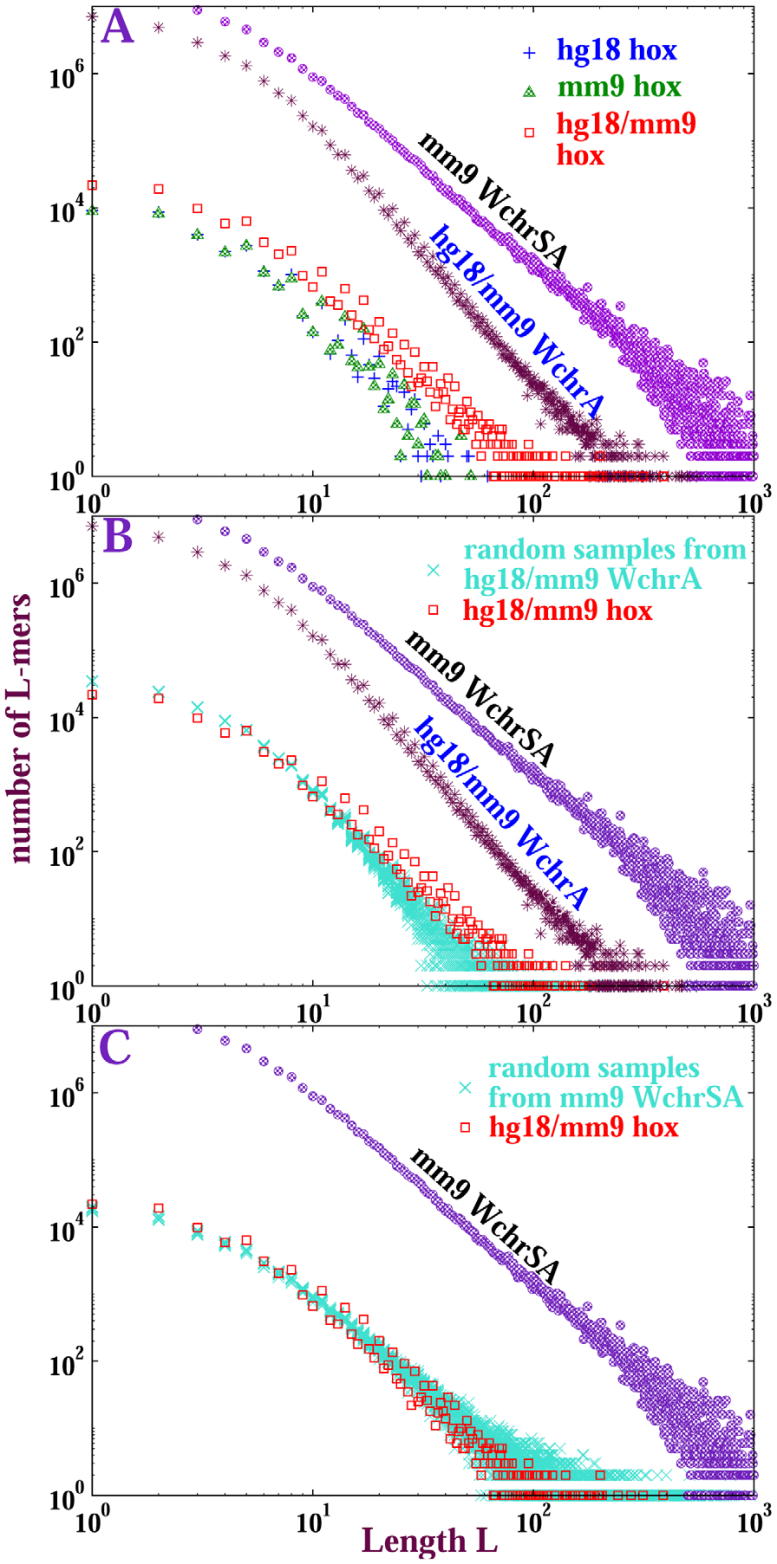
Length distributions of exact matches from Hox gene sequence alignments. The reference distributions are: (1) **mm9 WchrSA**: *self*-alignment of all Hox gene-containing mouse chromosomes (chromosomes 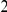, 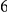, 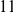 and 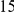); (2) **hg18/mm9 WchrA**: hg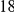-mm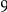 *inter-species* alignments among Hox gene-containing chromosomes only. Symbols in subfigures: Red squares (hg18/mm9 hox): Hox gene CMRs from hg18/mm9 alignment; Green triangles (mm9 hox): Hox gene CMRs from mm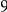 self-alignment; Blue pluses (hg18 hox): hox gene CMRs from hg18 self-alignment; Turquoise crosses: lay out of 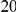 sequence sets, each randomly sampled from respective parent distributions: hg18/mm9 alignment in subfigure B and mm9 self-alignment in subfigure C. In all these random samples, Hox gene sequences have been excluded. Each sample contains the same total number of matched bases as Hox gene alignments from hg18/mm9 CMRs.

Figure 7 displays length distributions of Hox CMRs retrieved from self-alignment and inter-species alignment of human and mouse whole genomes. For exact matches, the length distributions are approximately algebraic with slopes near 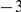. Of the sequences composing them, 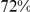 (respectively, 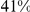, 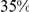) of the Hox CMRs longer than 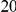 bases in mouse self-alignment (respectively, human self-alignment, human-mouse alignment) are protein-coding. Nevertheless, these distributions appear roughly homothetic (similar in shape) to full whole-chromosome self-alignment as seen in Figure 7 A, and also to mouse whole-genome self-alignment (data not shown).

The Hox CMRs from human/mouse inter-species alignment show similar length distributions to those retrieved from mouse or human self-alignment. The length distribution of these Hox CMRs seems homothetic to the self-alignment, but not to the inter-species alignment.

To quantify these apparent similarities, we generated sets of sequences for comparison by randomly sampling from human/mouse alignment, excluding Hox genes. Each sample is chosen to contain the same total number of bases as in the human-mouse Hox CMRs. Twenty independent sets were sampled, yielding length distributions homothetic to their parent distributions but *not* to the human-mouse Hox gene alignment length distribution (Figure 7 B). On the other hand, as shown in Figure 7 C, the distributions from human/mouse Hox gene alignments coincide with those of random samples from mouse whole-chromosome self-alignment, and they are all homothetic to the mouse self-alignment.

For A = G/T = C runs, the Hox-gene alignment exhibits properties identical to those for exact matches (Figure *S9* A–C); because they are so poorly sampled, we can't say the same about alignment block lengths and indel-terminated runs. In the right column of Figure *S9* (subfigures D–F), length distributions of contiguous indels are observed to parallel one another, and random sampling yields distributions homothetic to their parent distributions (see Figure *S9*).

For nearly all the alignment fragments that overlap Hox gene sequences in whole-chromosome self-alignments, both query and target were found to overlap a Hox gene. Very few pairs were aligned between a Hox gene and a gene not in the Hox gene set. As shown in Figure *S8*, it is always the same region of the 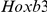 gene that is matched to another Hox gene; Hox-gene alignments seed at this high-similarity region and are extended into its neighborhood. This high-similarity region contains the coding sequence for the homeobox domain.

In summary, mouse/human alignment indicates that Hox genes are atypical of conserved sequence genome-wide, because they exhibit 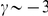 rather than 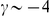. Since ultraduplicated sequence represents less than 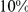 of these genomes, it is plausible that they contribute insufficiently to the mouse/human alignment to alter the genome-wide 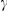 from 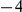. Within Hox genes, it appears that 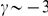 is independent of whether the aligned sequences are protein-coding, consistent with the hypothesis that ultraduplication is a neutral process.

It is worth observing that the value 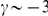 has in principle nothing to do with the fact that a codon consists of 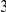 nucleotides: 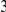 bases is the length of a codon, but 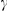 is dimensionless.

## Discussion

### Power-laws

Algebraic (or power-law) distributions are ubiquitious in complex systems; e.g. the connectivity of the world-wide web; the cooperation network of actors and actresses [13]; CD sales rank; the number of articles with a given number of citations [14]; the number of words with a given number of occurrences in a genome or text (Zipf law). Power-law distributions in biology are most commonly, as in these examples, ranked lists; such phenomena have been observed at different levels of organisation (Interpro families, protein superfamilies and folds, pseudogene families and pseudomotifs) and for a variety of attributes, including function, interaction and expression level [15]; however, in these contexts their biological significance may not be readily apparent. Their popular interpretation as “dominance of the very few” is in general either misleading or inaccurate.

The class of algebraic distribution analyzed in this paper and in our studies of sequence conservation is distinct from the class of examples mentioned in the last paragraph; rather, it is typical of those more often observed in the physical sciences, for example at critical points of second-order phase transitions. We explore the numbers of sequences duplicated within a genome (or conserved between two genomes) as functions of their lengths. Length is a geometrical quantity, with a natural metric interpretation in terms of physical distance measured in nucleotides or nanometres; as Mandelbrot observed in the 1950s, this geometric content distinguishes fundamentally the distributions we study from ranked lists [16], [17]. Because length is a dimensional quantity, it would not be expected that these distributions could be derived from Zipf distributions; independent information would be required.

A set of conceptual tools for analyzing geometry-based distributions was developed in the physical sciences starting in the middle of the twentieth century [18]. Recent popular guides to characterizing the forms of distributions steer clear of examples that are geometry-based, focusing instead primarily or exclusively on ranked lists of marginal relevance to this study [19]. In particular, physical sciences concepts stress that *any algebraic form applies strictly only in the limit of diverging system size* (genome length 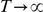 in the current context) – e.g., asymptotically in a thermodynamic or continuum limit [20]. For finite system sizes, a purely algebraic form expected to represent at best an approximation to the real world; ultraviolet (short length, high energy) and infrared (large scale, low energy) corrections are inevitable, and a satisfactory theory ought to account for them.

Nevertheless, the dynamics behind ranked lists on the one hand and scaling phenomena in the physical sciences on the other, can both be governed by correlation. For example, the observed power-law distribution of the number of papers with a given number of citations can be explained by preferential attachment, a stochastic model in which new citations accrue in proportion to the number of previous citations [14], [21]. We can't infer therefore that citation is a purely stochastic process, but we might anticipate that such correlated randomness needs to be corrected for when interpreting citation counts. Similarly, one expects that a sequence physically linked to neighboring elements under selection is itself more likely to be conserved, and we anticipate the need for an analogous correction when interpreting its conservation.

Ref. [19] observed that linearity on a log-log plot is insufficient to infer a power-law form; in addition strong curvature on a semi-log plot ought also to be observed; if it is not, then an exponential form can't be excluded. We have plotted all our data on semi-log axes, either as insets of the log-log plot, or if they don't fit there, in the manuscript or supporting data. Fitting to a numerical dynamical model also supports our interpretation; an example is illustrated in *S1* although the model is described in detail elsewhere.

Ranked lists of occurrences of words of fixed length have been studied in genomes and texts [22], [23]; their forms may often be algebraic; however, as we have mentioned above there is no natural physical metric – these distributions are of Zipf type, and their proper interpretation remains elusive.

### Long-range correlations in genome sequences

Algebraically decaying two-point base correlations in genome sequences have been studied intensively since the early 1990's; see Ref [24], [25]. for thorough reviews of these efforts. These correlations appeared for a while as if they might implicate a non-local component of genome sequence evolution. In this manuscript, by the term “local” we refer to “local with respect to the linear chromosome sequence.” Obviously, higher-order chromosomal structure could lead to effects that are local in space, but non-local on the genome sequence; such non-locality was embodied in one of Stanley's early models [26] as internal looping of a self-avoiding polymer [27], leading to random deletions and insertions of sequence tracts with probability 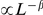, 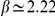.

Two distinct proposals for the origin of non-locality, one by Grosberg and co-workers [28] and one by Stanley and co-workers [19], suggested that the non-locality arose from higher-order chromosomal structure; the former as a collapsed polymer globule, the latter as a self-avoiding (non-Gaussian) polymer. Analytical derivations of sequence correlations as a function of the loop length distribution exponent 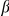 were obtained within a simpler “generalized Levy walk” model [29]. These proposals appear to have been largely superseded by an alternative mechanism, the Li expansion-modification model [30], which accounts for non-locality of the static correlations by purely local genome growth dynamics. Exponents for the Li expansion-modification models of genome growth have been analytically derived [31]. More recently, Stanley and co-workers have proposed an “unequal crossing-over model” to explain algebraic length distributions of dimer tandem repeats [32]; however, these “simple” repeats comprise a negligible contribution to the sequences we study here. None of these models, in the forms originally proposed, generate algebraic duplication length distributions as we defined them here.

These mechanisms are – all of them – neutral, as they do not depend on sequence functionality – no phenotype is expressed to be selected for or against. It was not apparent that any observables could distinguish among them; however, the duplication distributions described here would seem to be inescapably non-local. The duplication length distribution turns out to be a characterization of genome sequences independent of, and orthogonal to, these long-range (spatial or positional) correlations, because positional information, such as correlation of locations of duplications within the linear genome sequence, has no direct impact on the duplication length distribution. That is, a tandem duplication is not counted any differently than two copies of a sequence separated by a distance on order of chromosome length.

We have demonstrated elsewhere numerical evidence that the expansion-duplication models yield exponential decay of duplication lengths, suggesting that it is an orthogonal phenomenon. The algebraic decay of ultraconserved sequence lengths [8] is similarly independent of base-base correlations, because this decay depends only on the evolutionary distance between genomes, and minimally on the genomes themselves: e.g. it is a property of pairs of genomes, not of individual genomes, and seems to show some universality.

### Comparative Genomics and Ultraconservation

The field of comparative genomics – of pivotal importance to medicine, biotechnology and the biosciences – relies on the inference of function from sequence conservation. Its premise is that selective adaptation acts on neutral (sequence) variation. If for any given sequence, it can be established that its conservation among diverse species is improbable on neutral sequence variation (or “drift”) alone, then selection on the function of the given sequence is inferred *de facto*. This premise underlies the “conservation tracks” on the genome browser at UCSC, for example. Consequently, the choice of a model for neutral drift can have a major impact on the computational inference of whether or not a sequence is functional.

The study described in this manuscript was motivated by our efforts to explain heavy, approximately algebraic tails in the length distributions of sequences strongly conserved among diverse species [8]. Indeed many of the features observed here for duplications *within* genomes parallel those of sequences conserved *between* genomes [33].

In the mid-1990's Brenner and co-workers sought long sequences shared among fragments of the pufferfish, mouse, and human genomes, subsequently demonstrating their activity as enhancers *in vivo* [34], [35]. More recently Bejerano *et al.* reported “ultraconserved” elements shared by human, mouse and rat genomes [36]: genomic subsequences that are identical among these three genomes over lengths exceeding 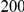 contiguous nucleotides; few of these elements were annotated at the time, but since then enhancer activity has been observed in more than half of the longest of these sequences.

The potential interest of shared long sequences of high identity is that – provided the genomes have diverged sufficiently – it is believed that such similarities are unlikely to have evolved by chance. In particular, under an independent-site substitution model, such long sequences of identity among these genomes are astronomically improbable in the absence of negative selection. Their occurrence is therefore attributed *de facto* to selection on function.

**Figure 8:**
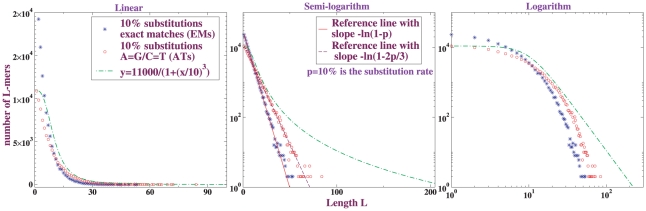
Local models yield exponentials. Length distribution of exact matches (blue) and A = G/C = T matches (red) in the alignment of a random sequence against a randomly point-substituted version of itself. The percentages indicate the rate of substitution per base.

Independent-site substitution models form the basis for inference of selection from sequence conservation [37]; correlations are explicitly assumed negligible [38]. Their virtue is that they and their close relatives are *local* models; conservation at one genomic location is assumed not to affect conservation at distant genomic locations. In the absence of selection, local models must yield shared sequence length distributions of the form shown in Figure 8: exponential (or geometric), with a slope on a semi-log plot that depends on the details of the model [24], [39].

This exponential character is not altered by uncorrelated positional variation of substitution rates, since they combine multiplicatively. Suitably correlated positional variations of substitution rates could in principle generate algebraic behavior – but correlations of the rates themselves would then need to be long-ranged. In short, if genomes evolved independently via local substitution and short indels only, the lengths of the sequences conserved among them should decay exponentially, absent effects of selection.

Nevertheless, it has long been appreciated that certain routine genomic processes, recombination in particular, are non-local in their impact. These processes are regarded as neutral insofar as they are not directly influenced by functionality, if any, of the sequences involved. We've argued that the most important implication of the data on strongly-conserved sequence elements is the failure of the independent-site substitution model for their proper interpretation [33]. In particular we observed in 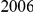 that the length distribution of these conserved sequences takes a distinctive algebraic form that – on its face – invalidates an independent-site substitution model [8]. It is difficult to understand how such a distribution could be derived from a dynamics that does not involve strong and systematic effects of conservation at one location on conservation at distant locations.

One possible origin of these effects is selection for function; however the largest contribution to exactly conserved sequence, both in bases and raw counts, from human/mouse (or human/mouse/rat) alignment arises at lengths not much longer than 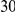 bases, overwhelming the contribution of lengths greater than 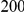 bases by orders of magnitude. A scramble to characterize the function and evolution of these short elements would indicate that this explanation is taken seriously.

Another explanation, whose impact must be disentangled from that of selection, is that the baseline neutral model (or “null model”) for genome sequence evolution against which conservation implies selection, ought to properly incorporate non-locality. Since the inference of selection in comparative genomics relies exclusively on local independent-site substitution models as “null models”, it should not be unexpected that the interpretation of conservation would be contaminated by non-local effects.

Thus, although one explanation for high identity is that sequence variation is constrained by selection for function [37], it has been understood for many years that certain kinds of neutral processes can also reduce sequence variation, among them selective sweeps, background selection, and hitchhiking – processes that act on physical linkage of alleles via recombination [40], [41].

Algebraic distributions of conserved sequence lengths turn out to apply far more generally than ultraconservation. In Ref [8]. we reported the scale-invariant structure of pairwise exact-matches (perfectly conserved sequences, or PCS) and reduced stringency-matches between distant genomes. The length distributions of PCS in both human/mouse whole-genome intersection and alignment exhibit algebraic forms with a slope close to 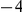 on a log-log plot, except at very short lengths. Human/mouse/rat whole-genome intersection and alignment display the same form, with the so-called ultraconserved sequences composing only the extremity of the algebraic tail; there is no separation of scales and the principal contribution to the algebraic tail comes from much shorter sequences.

We demonstrated that an algebraic length distribution with exponent 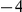 is a feature of intersection and alignment between a wide variety of eukaryotic genomes as distantly-related as human and sea urchin, whereas an exponential distribution is typical of closely-related genomes [8], [33] (see for example Figure 1 B; see also the section “Bergman and Kreitman” in the Text *S4*). Relaxing the stringency of matching by, for example, tolerating A/G and C/T mismatches (A = G/C = T runs), terminating a run of contiguous aligned sequence only at an indel, or treating an entire alignment block as a matching run, yields a distribution with approximately the same shape as PCS. Prokaryotic genomes display qualitatively similar behavior, although the exponents vary over a wider range.

Developments in population genetics over the last twenty years have lead to an increasing appreciation of the role of neutral DNA recombination processes in shaping genome sequence, under the banner of “concerted evolution,” although quantitative characterization of these processes is an currently area of intensive activity.

### Some conjectures on mechanism

Finally, we speculate on the mechanism of generating a power-law source of duplication lengths.

#### Eichler's mechanism

Eichler characterized segmental duplications and their flanking sequences in humans in detail, and observed that segmental duplications in humans are often bracketed by *Alu* SINE sequences [42]. His definition of segmental duplication differs considerably from ours; by our more pristine definition, human segmental duplications are, with respect to their length distributions, quite typical of genome-based life forms.

Nevertheless, the notion that ultraduplication may be mediated by a form of transposable element has certain attractions. In particular, although some classes of transposable element are strictly constrained to narrow ranges of insert length, others serve as junctions that invoke the action of non-specific recombination mechanisms on sequences that they bracket. These recombination mechanisms can be sensitive primarily to the local structure of the junction, and not as much to global features such as the length of the insert. Thus, insert lengths would not be dictated by the functionality of the insert sequence, but rather by global considerations, such as the higher-order structural organization of the genome in space or scaling behavior originating in polymer physics [26].

#### Rokhsar's proposal

Rokhsar suggests that a scale-invariant distribution of duplicated sequence lengths within a common ancestor induces correlations in recombination events subsequent to speciation by providing (common ancestral) homologies as substrates for recombination in descendents.

The scale-invariant distribution of shared sequences among descendents (e.g. of the ultraconserved sequences) therefore emerges from the scale-invariant distribution of the duplications in the common ancestor. The mechanism of recombination is not specified, but homologous recombination is presumably one candidate.

#### Brenner's conjecture

Brenner conjectures that the power-law may be generated by “molecular drive” (also known as “meiotic drive” or “concerted evolution”) [4]; specifically by gene conversion. The parallel shift of the A = G/C = T distributions versus the exact match distributions suggests a potential role for GC-biased gene conversion [43] coupled with an algebraic distribution of gene-conversion tract lengths, as a possible mechanism for generating isochores. This possibility is under investigation.

#### MEPS

A finite-order Markov model can't be the source of other than an exponential distribution of sequence lengths (its memory must be at least as long as the tail of any algebraic distribution it generates). One natural candidate for a mechanism with a long memory arises from the MEPS (minimum efficient processing segment), the shortest stretch of strict sequence identity necessary for recombination to proceed at significant rates [44], [45]. This process has the flavor of nucleation, and its subsequent extension provides an ingredient for suppressing locality and exponential decay of duplication or recombination lengths: at any given time during duplication, the probability of extension is likely to depend on the length of the sequence already matched before that time (the longer the match, the less likely that the responsible protein complex falls off and terminates). Furthermore, the lengths of recombining sequences under homologous recombination depends on the homology between the sequences, in a manner that has so far been investigated primarily on a “mean–field” (e.g. % similarity) basis. Both MEPS and homology dependence could yield instabilities in the dynamics of evolving genome sequence.

#### D-loops

D-loops (displacement loops) are intermediates in the recombination process that can be directly observed by electron microscopy [46]. They represent the DNA segment displaced by the invading strand. The algebraic tail described here could arise from the distribution of D-loop lengths (presumably including intermediates that abort without yielding recombinants).

#### Other considerations

The action of recombination on genome sequences is itself likely to be under strong selection, while at the same time subject to physical constraints that reflect the global geometry of a genome. It may be that genomic sequence data will enable a tighter characterization of recombination; for example, what are the properties of an optimal recombination mechanism? We expect that gene conversion tract lengths and duplication-length distributions likely to feature strongly in such a characterization.

### Conclusion

We previously demonstrated that strong sequence (including ultra-) conservation exhibits an algebraic length distribution, yielding a heavy tail of conserved sequences with no evident separation of scales. This conservation of the longest of these sequences is customarily attributed to selection for function; however, we have argued that it is attributable at least in part to the impact of neutral processes of linkage and recombination. Such an argument is – naively – implausible in the absence of evidence that recombinative processes can by themselves generate an algebraic length distribution. This manuscript demonstrates that segmental duplication processes do indeed generate an algebraic length distribution, not only globally but locally as well. A direct connection between these two algebraic length distributions remains to be drawn.

## Materials and Methods

### Self-alignment

We studied several eukaryotic and bacterial genomes. Eukaryotic genomes are typically packed with repetitive sequence, close to half of the human genome, for example, reducing the effectiveness of whole-genome alignment methods dramatically. Repeat-masking is a heuristic method for tagging simple repeats, certain complex interspersed repeats, and sequences similar to them (http://repeatmasker.org). Whole-genome alignment of eukaryotic genomes has so far relied on their removal via “repeat-masking” before alignment, although some of them are heuristically reintroduced after the alignment of repeat-free sequences. Soft-masked sequences were retrieved from the Ensemble databases (http://uswest.ensembl.org/index.html) through the Ensembl APIs. Bacterial genomes can be aligned without repeat-masking; we used unmasked sequences retrieved from the NCBI ftp server (ftp://ftp.ncbi.nlm.nih.gov/genomes/).

Sequences were aligned by Blastz and the output translated into Axt format to produce a “Blastz-Raw” alignment. Further processing by Chain and Net yields respectively “Blastz-Chain” and “Blastz-Net” alignments respectively. Chain primarily reorganizes fragments generated by Raw and drops those with low similarity scores; Net filters the chained alignments to retain only those scoring highest for similarity and concatenates them into a single long chain [47]. We study the outcome of each of these three stages of alignment and make comparisons among them. All necessary executables can be found at the UCSC website (http://hgdownload.cse.ucsc.edu/downloads.html), and a convenient alignment procedure may be found at: http://genomewiki.cse.ucsc.edu/index.php/Whole_genome_alignment_howto (Websites accessed on 2011 Mar 16th).

In order to establish that our primary observations are not artifacts of the alignment algorithms, we also performed some of our alignments with another software tool, Mummer (http://mummer.sourceforge.net/), and compared its output to that of Blastz. Mummer's procedure differs from Blastz's; for example, its first step involves an exhaustive all-on-all search for exact matches (“seeds”), whereas Blastz invokes a heuristic search for seeds that needn't be exact matches. They also differ in how they treat repeat-masked sequence and extend the seeds. For our purposes, it turns out that the outcome of Mummer generally tracks that of Blastz very well, suggesting that artifacts of alignment do not account for our observations.

The following genomes and chromosomes sequences were aligned: the eukaryotes *Homo sapiens* (human), *Mus musculus* (mouse), *Pan troglodytes* (chimpanzee), *Gallus gallus* (chicken), *Tetraodon nigroviridis* (freshwater pufferfish), *Drosophila melanogaster* (fruit fly), *Saccharomyces cerevisiae* (yeast), *Caenorhabditis elegans* (worm) and *Oryza sativa* (rice), whose soft-masked sequences were retrieved from Ensembl Core databases version 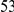 (except that for *Oryza sativa*, we use version 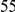); and the prokaryotes *Anabaena variabilis*, *Cyanothece PCC 7424*, *Cyanothece PCC 8801*, *Gloeobacter violaceus*, *Salmonella enterica Choleraesuis*, *Escherichia coli 536*, *Nostoc punctiforme PCC 73102* and *Vibrio cholerae M66_2*, downloaded from NCBI.

### Special features of self-alignment: self-hits and reciprocal pairs

Self-alignment differs from inter-genome alignment in two important respects:

1. Self-hits: Since any sequence matches itself perfectly, there is in principle always a “perfect chain” in a self-alignment: the whole chromosome. In practice, repeat-masking and other details of the alignment procedure break this perfect chain into perfectly-matching sub-chains (referred to here as “self-hits”) that lie exactly on the diagonal of a dot plot: they are identifiable because they derive from the same location in both the query and the target. In this sense, they are trivial and they are not of primary interest here. For Blastz-Chain and Net alignments, the perfect chain has the highest score and will suppress any other potential contributions to the alignment; therefore, we eliminate the self-hits from Raw alignment before further processing. Similarly, the Mummer alignment algorithm eliminates self-hits before assembling exact matches into chains.
2. Reciprocal pairs: Among aligned fragments that are not self-hits, there arise so-called reciprocal pairs: pairs of aligned sequences in which the query sequence of one is precisely the target sequence of the other and *vice versa*, so that they are actually equivalent to each other. In our calculations we count only one contributor from each pair.

## Supporting Information

Figure S1 **Schematic map of the CMRs subject to different matching stringencies.** We chose a representative alignment block from the Blastz-Raw self-alignment of *Anabaena variabilis* whole genome and highlighted the CMRs according to each of the different matching criteria. Bacterial genomes are relatively small and their CMRs are short enough that it's possible to achieve single-base resolution in a legible figure. Each dash “-” in the figure corresponds to one indel (a single base insertion or deletion). The rectangles and arrows indicate the nesting; a single CMR at relaxed stringency may contain several CMRs at greater stringency. From the top down, as the matching criterion becomes tighter, the CMRs are deconstructed into finer sequence elements.
(EPS)

Figure S2
**Length distributions counted by different approximate matching criteria** in mouse chromosome 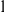 self-alignment. (1) A = G, C = T; (2) A = C, G = T; (3) A = T, G = C.(EPS)

Figure S3
**Length distribution of the self-alignments of a real genome and three synthetic sequences:** (a) *Anabaena variabilis* whole-genome self-alignment; (b) Self-alignment at steady-state of a scale-free duplication dynamics [10]; (c) Self-alignment of a random sequence after single whole-genome duplication followed by 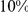 random single-base insertion/deletion; (d) Self-alignment of *Anabaena variabilis* whole-genome following 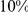 random single-base insertion/deletion. Total sequence length is kept fixed at around *Anabaena variabilis* whole-genome sequence length for (a)–(d).(EPS)

Figure S4
**Dot plots of self-alignments of mouse chromosome** 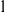 **and** ***Anabaena variabilis*** **genome.** Alignments are computed by Blastz-Raw, Blastz-Chain, Blastz-Net, and Mummer respectively. Blastz-Raw and Chain yield almost identical in dot plots, which are apparently denser than Blastz-Net and Mummer.(EPS)

Figure S5 **Length distributions of exact matches in Blastz-Raw self-alignments for each mouse chromosome.** Log-log plots, with semi-log insets.
(EPS)

Figure S6
**Length distributions and dot plots for CMRs from forward and backward alignments.** Two different panels for mouse chromosome 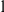 and *Anabaena variabilis* genome respectively. Alignments by Blastz-Raw.(EPS)

Figure S7
**Length distributions of contiguous aligned/unaligned bases in the projection onto the chromosome of self-alignments.** [(a), (b)] for mouse chromosome 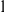 and [(c),(d)] for *Anabaena variabilis* whole-genome. In order to confirm that the aligned sequences are not randomly distributed in the genome, we placed randomly onto the chromosome a set of sequences with the same length distribution as the aligned sequences, computed the length distribution of the complementary set, shown by the curves labeled “random”, which are clearly exponential. Alignments by Blastz-raw.(EPS)

Figure S8 **Schematic map of Hox gene sequence alignments.** From the whole-genome self-alignment of mouse, we retrieve all aligned fragments for which both the query sequence and the target sequence overlap with a Hox gene (not necessarily the same Hox gene or the same chromosome). Then we cut out the overlapping regions and extract the CMRs. This figure shows some fragments from the *Hoxb3/Hoxb5* alignment. The ellipses “….” represent outlying parts of the genes that are not pictured here.
(EPS)

Figure S9
**Length distributions of A = G/C = T runs and contiguous indels from Hox gene sequence alignments.** The reference distributions are: (1) **mm9 WchrSA**: *self*-alignment of all Hox gene-containing mouse chromosomes (chromosomes 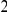, 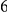, 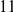 and 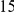); (2) **hg18/mm9 WchrA**: hg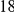-mm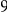 *inter-species* alignments among Hox gene-containing chromosomes only. Symbols in subfigures: Red squares (hg18/mm9 hox): Hox gene CMRs from hg18/mm9 alignment; Green triangles (mm9 hox): Hox gene CMRs from mm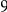 self-alignment; Blue pluses (hg18 hox): hox gene CMRs from hg18 self-alignment; Turquoise crosses: lay out of 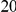 sequence sets, each randomly sampled from respective parent distributions: hg18/mm9 alignment in subfigure B and mm9 self-alignment in subfigure C. In all these random samples, Hox gene sequences have been excluded. Each sample contains the same total number of matched bases as Hox gene alignments from hg18/mm9 CMRs.(EPS)

Text S1 **Scale-free duplication dynamics.**
(PDF)

Text S2 **Comparison among different alignment methods.**
(PDF)

Text S3 **Homogeneity of power-law length distributions among different subsets of the alignment.**
(PDF)

Text S4 **Bergman and Kreitman.**
(PDF)
