# Supplementary material for: Algebraic Distribution of Segmental Duplication Lengths in Whole-Genome Sequence Self-Alignments
Source: PLoS One. 2011 Jul 14;6(7):e18464. doi: 10.1371/journal.pone.0018464 (PMC3136455; doi:10.1371/journal.pone.0018464)
Supplement: Text S1 — Scale-free duplication dynamics. (PDF) [file pone.0018464.s010.pdf]

Text *S1*. Scale-free duplication dynamics

Elsewhere, we have described a duplication model for a fixed-size genome in which duplication lengths L are obtained from a power-law source distribution with exponent γ: $f(L) \propto L^{\gamma}$. A sequence of length L is chosen from the genome and substituted for sequence at a randomly chosen location elsewhere in the genome. At long times, a steady state is achieved wherein the distribution of duplicated sequence is found also to obey a power-law, but with an exponent close to $\gamma - 1$. This difference between source duplication length distribution and effective duplication length distribution can be understood as arising from the interaction of duplicated sequences with one another: overlap leads to truncation. Numerical simulations have been reported, but analytical confirmation has also recently been obtained.

Figure *S3* (a) shows CMRs from *Anabaena variabilis* self-alignment, for reference; (b) shows CMRs from a self-alignment of sequence of the same total length T as *Anabaena variabilis* from the steady state of our scale-free duplication model with source $\gamma = -3$; (c) shows CMRs from a random sequence of length $T/2$ that has been duplicated once to yield a sequence of length T, and then subjected to $T/10$ random one-base insertions and deletions. Finally, (d) shows CMRs from *Anabaena variabilis* sequence subsequent to $T/10$ random one-base insertions and deletions. A reference line of slope -4 is drawn for reference; the plots are log-log with semi-log insets.

To generate Figure *S3* (b), L was chosen from a source distribution of the form $1/[1+(L/a)^{-\gamma}]$ with $a = 16$ and $\gamma = -2.4$. The sequence of length L was inverted (reverse-complemented) with probability one-half before resubstitution into the genome. Single-base substitutions were applied randomly in the genome at a rate of $\mu = 2 \times 10^{-5}$ per base per iteration. As in real genomes, the point mutations are such that A⇔G and T⇔C are more likely than other substitutions; within our model, some such asymmetry is essential to obtain the observed displacement of the exact-match versus the A=G/C=T curves in the algebraic regime.

Among these four subfigures, the shapes of the curves bear comparison. The exponential forms in (c) are expected theoretically for uncorrelated indels, and are clearly distiguishable from the forms in (a) and (b). Subfigure (d) demonstrates that with the exception of the contiguous indel length distribution, exponential forms are obtained unless duplications occur on time scales comparable to uncorrelated mutation local. The steady-state of scale-free duplication dynamics yields power-laws in the limit of large T (data not shown), so subfigure (b) indicates very roughly the finite-size effects that would be anticipated for *Anabaena variabilis* size genomes. The model is an idealization, of course. In particular, it doesn't include autonomously replicating sequence elements, each of which has its own characteristic size and replication rate. Adding such elements to the model goes a considerable way towards reconciling the discrepancies in overall scale evident between (a) and (b); details will be given elsewhere.
