# Supplementary material for: Algebraic Distribution of Segmental Duplication Lengths in Whole-Genome Sequence Self-Alignments
Source: PLoS One. 2011 Jul 14;6(7):e18464. doi: 10.1371/journal.pone.0018464 (PMC3136455; doi:10.1371/journal.pone.0018464)
Supplement: Text S2 — Comparison among different alignment methods. (PDF) [file pone.0018464.s011.pdf]

Text *S2*. Comparison among different alignment methods

In Section ***"Insensitivity of the power-law to the alignment method"*** in the main text, we've observed that different comparison methods yield qualitatively consistent outcomes. The form of the length distribution is insensitive to the method; any differences are for our purposes insignificant.

In Figure 4 of the main text, the similarities among length distributions obtained by different *alignment* methods are apparent on the log-log plot. Also illustrated there is the outcome of self-*intersection*, which corresponds to the alignment at sufficiently large scales; below those scales, random matches produce a deviation from a power-law that is exponential and is suppressed here following [8]. But within those scales, it is evident that distributions from self-intersection and self-alignment are not readily distinguishable. The Chain alignment almost overlaps with the Raw alignment throughout, except for the contiguous indels; Chain discards large contiguous indels from Raw. Raw and Chain both contain overlapping alignments – multiple fragments of query sequence can be aligned to the same fragment of target sequence. In contrast, the Net alignment filters the alignments, keeping only the "best" chains for each target. Mummer eliminates most of the overlapping alignments in its very first stages. The curves for Blastz-Net and Mummer overlap partly, and both of them have fewer CMRs than Lastz/Blastz-Raw and Chain, as recapitulated by the dot plots shown in Figure *S4*, where the dots are sparser for Blastz-Net and Mummer.

Mummer alignment differs from Lastz/Blastz alignments for our purposes primarily in the following two respects:

(1) Although Lastz/Blastz alignments can't be seeded within a repeat-masked region, a seed can subsequently be extended into such regions. Mummer, on the other hand, implements no special mechanism to process soft-masked sequences, and discards hard-masked sequences. Therefore, Mummer alignment applied to hard-masked sequences yields fewer CMRs than Blastz-NET.

(2) Mummer begins with an exact-match search and extends the alignment after filtering chains, so that it identifies fewer CMRs on short scales.

These differences don't affect the observation that all the distributions are qualitatively power-law, suggesting that the power-law is an intrinsic property of the CMRs and does not depend on the alignment algorithm. Since distribution shapes don't depend on the alignment algorithm, we subsequently apply solely Blastz-Raw for our alignments. We choose Blastz-Raw as a representative method because it's the simplest alignment method and it generates the greatest total quantity of aligned sequence. We don't claim that any one of these alignment methods is "better" than any of the others – for a given application, each method is likely to show its own set of advantages and drawbacks. For our purposes, it is apparent that there is not much distinction among them.
