# Supplementary material for: Algebraic Distribution of Segmental Duplication Lengths in Whole-Genome Sequence Self-Alignments
Source: PLoS One. 2011 Jul 14;6(7):e18464. doi: 10.1371/journal.pone.0018464 (PMC3136455; doi:10.1371/journal.pone.0018464)
Supplement: Text S3 — Homogeneity of power-law length distributions among different subsets of the alignment. (PDF) [file pone.0018464.s012.pdf]

Text *S3*. Homogeneity of power-law length distributions among different subsets of the alignment

Having studied length distributions of the CMRs on chromosomal or genomic (e.g. global) scales, we now investigate *local* structure of the length distributions on mouse chromosome 1 by Blastz-Raw self-alignment.

i. Homogeneity between forward and backward alignments

Since DNA is a directed polymer consisting of two complementary strands, one can constrain the alignment to the two forward strands (*forward alignment*) or a forward strand and a backward strand (*backward alignment*). (Obviously, provided that the alignment method is invariant under taking the reverse complement of the query and target sequences, then forward versus forward is equivalent to backward versus backward, and forward versus backward is equivalent to backward versus forward). Kong *et al.* observed a "high-level" global inverse symmetry in a wide variety of genomes [*S*1], suggesting large numbers of inversions throughout the backward alignment. In order to compare the properties of these two subsets of alignment, we count the forward and backward alignments separately and illustrate their length distributions and dot plots in Figure *S6*. It turns out that both the length distributions and the dot plots are almost identical between them, implying that close to 50% of SD are inversions, presumably accounting for this high-level inverse symmetry.

In Figure *S6*, we can see that this observation applies equally well to both mouse chromosome 1 and *Anabaena variabilis* genomes; however, for *Anabaena variabilis*, a difference between forward and backward alignment is apparent in the structure of the dot plot. The diagonal band observed in each of the other dot plots is absent in Figure *S6* (d). The distance to the diagonal in the dot plot indicates the distance between two matching sequences, so that the density of the diagonal band indicates the frequency of local duplication. In mouse genome, this diagonal band is visible in both Figure *S6* (a) and (b): both equidirectional and inverted segmental duplications are equally likely to occur within the neighborhood of the original sequence; however, in *Anabaena variabilis* genome it seems that equidirectional duplication is more likely to occur in that neighborhood, but inverted duplication not so.

This phenomenon has also been partly elucidated by Kong *et al.*, where they studied the χ_i-matrix for a wide variety of genomes and investigated their local inverse symmetries (LIS). They found different types of LIS among genomes of eubacteria and archaea; however, high-level LIS is found essentially only in the off-diagonal regions. This observation is consistent with what we describe here.

ii. Projection of the dot plot

We investigate here (i) the fraction of the chromosome that self-aligns; and (ii) the spatial distribution of the aligned sequences on the chromosome. Because we want to count each location on the chromosome only once – even when sequence at that location occurs in multiple copies – we project the self-aligned fragments onto the chromosome. One can think of this process as projecting the dot plot onto one of its coordinate axes; the length

distributions of contiguous aligned bases (bases that are contribute to at least one aligned duplication) and contiguous unaligned bases (bases that are never part of any aligned duplication) are computed. Multiply covered locations are only counted once.

In Figure *S7* A, we can see for mouse chromosome 1 self-alignment, in both the forward and the backward subsets, that length distributions of contiguous aligned bases are roughly power-law throughout. Overlapping fragments are merged in the projection, so that the slopes of the distributions are a little smaller than those of the CMRs in the original dot plot. For contiguous unaligned bases, the distributions have heavy tails representing large fragments of low similarity in the genome that are discarded during the alignment process; nevertheless at small length scales, they are also roughly power-law. On the other hand, the contiguous aligned bases at short lengths originate primarily in high-similarity regions; "local complements" of the unaligned bases, they follow the same spatial arrangement and exhibit roughly power-law length distributions.

The power-law length distribution of the contiguous unaligned bases implies a non-random spatial arrangement of the segmental duplications, as the genomic complement of an algebraic distribution of sequence lengths is not necessarily algebraically distributed. If there were no correlation among their locations so that duplicated sequences were randomly arranged in the chromosome, the gaps between them (i.e. contiguous unaligned bases) ought to show an exponential length distribution. To confirm this expectation, we randomly arranged a set of fragments with the same length distribution as the self-aligned sequences in mouse chromosome 1 onto a one-dimensional interval of chromosomal length, and counted the length distribution of the contiguous unaligned bases. As seen in in Figure *S7* B, these distributions for both the forward and backward alignments are exponential. Therefore, in real genomes, locations of duplicated sequence are strongly correlated with one another.

Since mouse chromosome 1 has been repeat-masked before alignment, we repeat this calculation for the self-alignment of *Anabaena variabilis* whole genome, which is not repeat-masked. The outcome shown in Figure *S7* C-D is almost the same as Figure *S7* A-B for mouse. Although the distributions in Figure 2 B fluctuate strongly, the one-dimensional projections of the aligned pieces exhibit relatively smooth power-law length distributions.

For mouse chromosome 1 Blastz-Raw self-alignment after projection, the forward alignment is constituted by 6.1% of the chromosome (before repeat-masking); the backward alignment by 4.4%; 7.7% in total. For *Anabaena variabilis* genome, the corresponding values are 21.2%, 19.0%, and 27.7% respectively.

Supporting References

[*S*1] Kong S G, Fan W L, Chen H D, Hsu Z T, Zhou N J, Zheng B and Lee H C (2009) Inverse symmetry in genomes and whole-genome inverse duplication. PLoS One 4(11):e7553.doi:10.1371/journal.pone.0007553.
