# Supplementary material for: Algebraic Distribution of Segmental Duplication Lengths in Whole-Genome Sequence Self-Alignments
Source: PLoS One. 2011 Jul 14;6(7):e18464. doi: 10.1371/journal.pone.0018464 (PMC3136455; doi:10.1371/journal.pone.0018464)
Supplement: Text S4 — Bergman and Kreitman. (PDF) [file pone.0018464.s013.pdf]

Text *S4*. Bergman and Kreitman

An exponential distribution of contiguously matching runs of bases in alignment of non-coding sequence between two species of *Drosophilae* was observed by Bergman and Kreitman in 2001 [*S*2]; we find an exponential distribution between all closely-related genomes, irrespective of whether the sequence is coding or non-coding. In contrast to the algebraic distributions described here, it is fully consistent with mutation-drift models. They did not study more distantly-related genomes.

Bergman and Kreitman studied the properties of sequence divergence in noncoding regions of fruitfly by comparing 100 *kb* of sequences in promoter regions and introns of 40 genes of *Drosophila melanogaster* and *Drosophila virilis* [*S*2]. Their strategy (comparison of a variety of alignment methods) turns out to be very similar to our own here and in Ref. [7, 33] although the context is quite different. They claim that these two species of *Drosophilae* have a divergence comparable to that between human and mouse, and report that the length distribution of the ungapped noncoding blocks conserved between them fits a log-normal distribution. Clark observed in Ref. [*S*3] that their data fit well to a mutation-drift model in which the base substitutions between two genomes are randomly distributed with equal probabilities per site.

Theoretically, the outcome of such an uncorrelated random process should be an exponential distribution at sufficiently large lengths, and Clark's Figure 1 indeed appears exponential. We verified this directly by aligning two sequences differing by p substitutions per base, and computing the slope of the distribution of perfectly-conserved sequence runs on a semi-log plot, which turns out, as expected, to differ insignificantly from $-\ln(1-p)$; $A = G/C = T$ matching runs show the expected slope $-\ln(1-2p/3)$, as shown in Figure 8 in the main text.

Bergman and Kreitman's log-normal distribution has two parameters, and it is always possible to obtain better fits with two parameters than with only one. It might be that Bergman and Kreitman were also concerned about the fit at short lengths (which is not relevant for our purposes), but that for longer lengths, the conserved blocks length distribution is fit just as well by an exponential. Clark also observes that *Drosophila melanogaster* and *Drosophila virilis* might not be as distantly related as Bergman and Kreitman believed; in our hands, all pairwise alignments of Drosophila subspecies with one another yield what appear to be plausibly exponential distributions of perfectly-conserved sequence lengths, similar in character to the human-chimpanzee distribution shown in Figure 1 B in the main text.

In summary, Bergman and Kreitman's outcome is fully consistent with our own; had they studied more distantly-related genomes, we anticipate that they would have also obtained distributions that are better fit with power-laws than with either exponentials or log-normals. As it is, Clark argued that their observations are consistent with a pure mutation-drift model, albeit with a uniform substitution rate that is less than the mean substitution rate of the genomes overall. In contrast, our observations are not consistent with pure mutation-drift.

Supporting References

[*S*2] Bergman C M and Kreitman M (2001) Analysis of Conserved Noncoding DNA in Drosophila Reveals Similar Constraints in Intergenic and Intronic Sequences. Genome Res 11:1335-1345.

[$S3$] Clark A G (2001) The Search for Meaning in Noncoding DNA. Genome Res 11:1319-1320.
